# The Regulatory Roles of Inflammation and Inflammasomes in Liver Diseases

**DOI:** 10.3390/ijms25189864

**Published:** 2024-09-12

**Authors:** Young-Su Yi

**Affiliations:** Department of Life Sciences, Kyonggi University, Suwon 16227, Republic of Korea; ysyi@kgu.ac.kr; Tel.: +82-31-249-9644

Inflammation is an innate immune response that protects our body from various pathogens and cellular dangers [1]; however, chronic inflammation, which is repeated and prolonged inflammation, is considered a critical risk factor for numerous diseases, including liver diseases [2,3]. An inflammatory response consists of two successive steps, the ‘priming’ and ‘triggering’ steps, which are the preparation and activation processes of inflammatory responses, respectively [4]. The cardinal feature of the priming step is the transcriptional activation of inflammatory molecules, while the key process of the triggering step is the activation of inflammasomes, which are intracellular protein complexes that provide a platform for inflammatory responses [4]. Previous studies have demonstrated that inflammation and inflammasome activation are implicated in the development of liver diseases [2,3,5], which proves that inflammasomes play a key role in inflammatory responses and liver diseases and could be potential targets for the development of novel therapeutics against liver diseases. However, the role of inflammasomes and their dysregulation during inflammatory responses and liver disease remain to be investigated.

The scope of this Special Issue included, but was not limited to, studies that explored the regulatory roles of inflammasomes in inflammatory responses and liver diseases, such as non-alcoholic fatty liver disease (NAFLD), non-alcoholic steatohepatitis (NASH), hepatitis, fibrosis, cirrhosis, liver injuries, and hepatocellular carcinomas (HCCs); this Special Issue also aimed to identify and validate novel targets regulating inflammasome functions and potential inflammasome-targeted therapeutics.

The review article by Yi highlights the regulatory roles of various flavonoids in caspase-11 non-canonical inflammasome-activated inflammatory responses. Previous studies have demonstrated the regulatory roles of flavonoids in canonical inflammasome-activated inflammatory responses and diseases [6,7]. Recent studies have also demonstrated the regulatory roles of flavonoids in non-canonical inflammasome-activated inflammatory responses and diseases. This review presents an overview of flavonoids and non-canonical inflammasomes and discusses the regulatory roles of various flavonoids such as luteolin, scutellarin, apigenin, epigallocatechin-3-gallate, quercetin, kaempferol, icariin, baicalin, morin, and naringenin in mouse caspase-11 and human caspase-4 non-canonical inflammasome-activated inflammatory responses; it also provides an overview of their role in multiple immunopathologies such as gastritis, sepsis, acute lung injury, pulmonary fibrosis, colitis, and renal ischemia/reperfusion injury. This review proves that flavonoids have anti-inflammatory effects and elucidates the use of flavonoid-based anti-inflammatory nutraceuticals in human diseases associated with non-canonical inflammasome activation.

The article by Qiu et al. investigates the proinflammatory roles of CXC motif chemokine ligand 5 (CXCL5) in Kupffer cell activation and the pathogenesis of drug-induced acute liver injury. This study demonstrated that CXCL5 inhibition mitigated drug-induced acute liver injury and ameliorated hepatocellular death in injured liver tissues by inhibiting inflammatory responses in mice. The in vitro study also revealed that CXCL5 increased drug-induced cytotoxicity in the hepatocytes co-cultured with Kupffer cells, and that CXCL5 inhibition reduced the LPS-stimulated inflammatory responses in Kupffer cells. This study proves that CXCL5 damages hepatocytes by inducing inflammatory responses in Kupffer cells.

The article by Beatriz et al. evaluates reactive nitrogen species (RNS) and NLRP3 inflammasome activation in the livers of lactating rats and their offspring when exposed to bisphenol F (BPF), a BPA analogue that is generated during the production of polycarbonate plastics. BPF significantly increased the expression of iNOS, an enzyme that produces RNS, activated NLRP3 inflammasome, and promoted the secretion of the pro-inflammatory cytokines IL-1β, IL-18, IFN-γ, and TNF-α in the lactating dams and their offspring when exposed to BPF. BPF exposure caused an increase in the production of RNS and pro-inflammatory cytokines, and activated NLRP3 inflammasome in the liver of lactating dams and their offspring. This study suggests that the industrial chemical BPF induces nitrosative stress and NLRP3 inflammasome-activated inflammatory responses in the liver, which causes hepatic injury.

The article by Cho et al. elucidates the regulatory role played by fatty liver neutrophil infiltration in nonalcoholic steatohepatitis (NASH) transition by focusing on interleukin (IL)-8, a key chemokine for neutrophil infiltration. This study reported an increase in IL-8 neutrophil infiltration and the development of liver injury in mice by regulating NADPH oxidase 2 complex. IL-8 induced the production of inflammatory cytokines by promoting macrophage activation, leading to the production of the factors responsible for fibrosis and hepatic stellate cell activation in mice. This study suggests that hepatic IL-8 promotes fatty liver progression to NASH by inducing neutrophil infiltration.

The article by Brujats et al. reports an observational case–control study that evaluates the clinical and immunological effects of SARS-CoV-2 on patients with advanced chronic liver disease (ACLD). The risk of liver decompensation was much higher in the SARS-CoV-2-infected ACLD patients compared to the non-infected patients. Furthermore, the serum IgG levels were significantly higher in the compensated SARS-CoV-2-infected ACLD patients compared to the decompensated patients. Additionally, the dysfunction of innate immunity in the patients with decompensated liver disease increased the risk of further decompensation following SARS-CoV-2 infection. This study suggests that patients with ACLD have a higher risk of decompensation following SARS-CoV-2 infection, which is possibly related to low-grade immune responses.

In conclusion, this Special Issue highlights the regulatory role of inflammasomes in inflammatory responses and human liver diseases. This Special Issue also demonstrated the underlying molecular mechanisms. We hope that this Special Issue provides critical insights regarding the regulatory roles of inflammation in human liver diseases and provides a basis for future scientific research exploring the role of inflammation in human liver diseases. We also hope that this Special Issue sheds light on the development of novel therapeutics for the prevention and treatment of various human liver diseases via the inhibition of inflammasome-mediated inflammatory responses.
